# Inside athletes' minds: Preliminary results from a pilot study on mental representation of doping and potential implications for anti-doping

**DOI:** 10.1186/1747-597X-6-10

**Published:** 2011-05-20

**Authors:** Andrea Petróczi, Jason Mazanov, Declan P Naughton

**Affiliations:** 1 School of Life Sciences, Kingston University, Penrhyn Road, Kingston upon Thames, KT1 2EE, UK; 2 Department of Psychology, The University of Sheffield, Western Bank, Sheffield, S10 2TN, UK; 3 School of Business, UNSW@ADFA, Northcott Drive, Canberra ACT 2600, Australia

## Abstract

**Background:**

Despite the growing body of literature and putative links between the use of ergogenic nutritional supplements, doping and illicit drugs, it remains unclear whether, in athletes' minds, doping aligns with illicit behaviour or with functional use of chemical or natural preparations. To date, no attempt has been made to quantitatively explore athletes' mental representation of doping in relation to illegality and functionality.

**Methods:**

A convenience sample of student athletes from a large South-Eastern Australian university responded to an on-line survey. Competitive athletes (n = 46) were grouped based on self-reported use as follows: i) none used (30%), ii) supplement only (22%), iii) illicit only (26%) and iv) both supplements and illicit drug use (22%). Whereas no athlete reported doping, data provided on projected supplement-, doping- and drug use by the four user groups allowed evaluation of doping-related cognition in the context of self-reported supplement- and illicit drug taking behaviour; and comparison between these substances.

**Results:**

A significantly higher prevalence estimation was found for illicit drug use and a trend towards a biased social projection emerged for supplement use. Doping estimates by user groups showed mixed results, suggesting that doping had more in common with the ergogenic nutritional supplement domain than the illicit drug domain.

**Conclusions:**

Assessing the behavioural domain to which doping belongs to in athletes' mind would greatly advance doping behaviour research toward prevention and intervention. Further investigation refining the peculiarity of the mental representation of doping with a larger study sample, controlling for knowledge of doping and other factors, is warranted.

## Background

One of the key constraints in designing effective anti-doping programs is the lack of conceptual clarity of the psychological mechanisms that influence doping behaviour. For example, preventing doping use in sport on the basis of fair play vs. cheating naturally lends to prevention and intervention programs that focus on the ethics of anti-doping and values, coupled with the consequences of being caught - not necessarily limited to sanctions but including dishonour, shame and guilt. Other programs may emphasise the potential hazards and detrimental health effects as consequences of doping use, which are omnipresent, independently from doping testing and sanctioning. What is largely unknown is what approach (if any) works as an effective deterrent [1]. Studies have investigated factors such as health [2], morality, sanctions if caught [3] and access [4] without making an attempt to construct a mental representation of doping in relation to morality and functionality. Evidence based on self reports has also been put forward suggesting a connection between prohibited performance enhancing and illicit drug use [5-10]. A potentially causal relationship between nutritional supplement (NS) use and doping has been suggested both theoretically [11] and empirically [12,13].

In the recent years, a number of athletes have talked publicly about their reasons and motives for doping use, contrasting perceived obligations and duty to perform well with guilt and the shame of lying. Studies conducted among professional athletes, particularly cyclists, offer valuable insight into how athletes perceive doping; and how this perception varies in different contexts [14-19]. In addition to the fact that many athletes consider doping as part of professional sport, most openly talk about experimentation with non-prohibited substances such as over-the-counter painkillers and non-steroidal anti-inflammatory drugs, caffeine and other non-prohibited stimulants [15,16]. Nutritional supplement use, which has been considered as a gateway to doping by many [12-14,20] is common among emerging and elite athletes [21-28] and has raised concerns on its own account owing to potentially harmful interactions from combined use and high dosage [29-32].

The question of whether doping behaviour has the character of illicit substance use, ergogenic substance use, neither or both has been recently raised in connection with anabolic steroids [33]. The ongoing debate is around whether the use of prohibited ergogenic substances aligns with behaviours associated with illicit substance use (e.g. psychoactive controlled drugs) or with nutritional (ergogenic) supplement use. Resolving which behavioural domain doping belongs in athletes' mind provides valuable insight for primary prevention activity. As doping has been categorised as an illicit (illegal) activity, it follows illicit drug models. Thus, the current anti-doping prevention follows typical demand control models seen for illicit drug use that focus on health education. It may be that the behaviour is functional with regard to its performance enhancing qualities. There is currently little in the way of ergogenic supplement primary prevention. Finally, doping may be an entirely new class of drug use behaviour, requiring a new set of primary prevention activities to be developed. The present research aims to inform which of these might be the case.

This pilot study is part of a larger research programme investigating social projection in various samples and context [34-37] and arose from some unexpected, but potentially far-reaching observations. The original aim of these studies was to investigate the utility of the biased social projection (False Consensus) as a proxy measure for substance use. Following from the findings [34,35], this study aims to compare and contrast social projection in different substance categories (i.e. supplements, illicit drugs and performance enhancing drugs) among athletes to quantitatively explore athletes' mental representation of doping in relation to illegality and functionality.

Previous results investigating social projection in performance enhancing and illicit drug use suggest that projected prevalence of doping and drug use was higher among self-admitted users respectively but absent for nutritional supplement use [34], and that this social projection was domain specific [35]. Domain specificity refers to an observed phenomenon that admitted doping use came with high estimations of doping use among other athletes with illicit drug use remaining unaffected; and conversely illicit drug users gave higher estimations of illicit drug use among others with estimated doping use remaining unaffected [35]. Although differentiating between cause and effect between social projection and behaviour in data from cross sectional research is impossible, the relationship is clearly present in self-reported data [34-37]. Interestingly, this phenomenon is only observable within the cognitively controlled information when athletes admitted the use of one or both of these drugs [36,37].

The importance of the social project lies with the question of whether an elevated and potentially distorted social projection leads to a congruent behavioural choice or resulted from it [38]. The fact that social projection aligns with self-reported behaviour but not necessarily with actual behaviour is intriguing, but more importantly it reveals something about athletes' cognitive processes relating to these substances. Thus, this may be used to gain insight into athletes' implicit mental representation of these, often concomitantly used, substances. Therefore by 'mental representation' we refer to an inferred psychological construct of which the athletes may be unaware. Congruently, the term 'athlete's mind' refers to the way athletes may subconsciously think about doping.

With the view of gaining some insight into athletes' implicit mental representation, we focused on social projection as an indirect indicator of mental representation of doping. Whilst recent evidence suggests that social projection cannot be safely used as a proxy for behavioural measures [36,37], declared social projections tells us something important about how doping is positioned in people's conscious mind. Descriptive norms are individuals' perceptions of how common a particular behaviour is. These norms are likely to be affected by some degree of projection (i.e. x% of athletes use doping). In particular, the projection may suffer from a social bias coined the 'False Consensus Effect' (FCE) [39] by which peoples' perception of their environment (including the behaviours of others) is distorted, thus resulting in a higher estimation.

The FCE is a perceptual bias where people who engage in particular behaviours tend to overestimate the proportion of the population who also engage in that behaviour. People who abstain either underestimate or correctly estimate prevalence. For example, marijuana users tend to overestimate the proportion of the population who use marijuana, and non-users are more accurate or underestimate [40,41]. A further characteristic is that the FCE is domain specific as it works within rather than across different categories or domains of behaviour [35]. Therefore, if doping was an ergogenic phenomenon, users of nutritional supplements should overestimate doping and *vice versa *(the positive case). Conversely if doping belongs to another domain, then the estimates of doping would occur independently of nutritional supplement use (the negative case). This suggests the relatedness of doping with either illicit or ergogenic substance use behaviours can be determined by emergent patterns of the FCE across behaviours.

**Figure 1:**
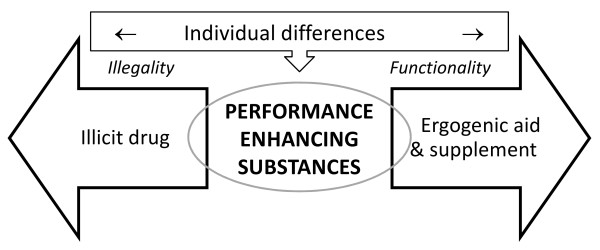
**Proposed schema of the mental representation of doping (prohibited performance enhancing substances)**.

In order to elucidate the peculiarities observed in the supplement-doping-drug triangle, this paper aims to test the domain specificity of doping relative to illicit substance and ergogenic nutritional supplement use via the FCE to explore whether it is an illicit or functional phenomenon. A proposed schema of mental representation of doping is depicted in Figure 1. Table 1 summarises the expected domain specific outcomes by user groups, assuming mutually exclusive categories (i.e. a nutritional supplement user is not a doping user or illicit drug user, etc.). In reality (and in our sample), it is likely that athletes use substances from two or even all three of these substance categories, thus making mixed categories with testable differences in their estimations of drug, doping and NS use. We hypothesise that commonality may emerge from the implicit mental representation. For example, athletes construct doping as illicit drug use and therefore athlete illicit drug users also overestimate doping. The legality of the substances may have a confounding influence, but this would have to be the subject of a future study following articulation of the concept offered here. A prediction in the NS use category is dependent on whether we believe that the FCE is purely retrospective justification, in which case, the projected figure should show no difference for two reasons: i) NS use is not a sensitive question and ii) we assume domain specificity (i.e. NS use is independent of doping and social drug use). However, if the FCE is, indeed, a normative belief, and the gateway theory stands, it would be logical to assume that NS users might give a higher estimation of doping users compared to their non-user counterpart. Although a similar argument could be put forward for the doping - illicit drug pair (i.e. use of one leads to the use of the other) but previous results indicated that this is not the case [35].

**Table 1:** Expected domain specific FCE schema

		Social projection (estimates)		
		**Illicit (social) drug**	**Performance enhancing drug**	**Nutritional supplement**
		
Illicit drug use	Yes	Higher than non-users' estimate	No difference	No difference
	No	Lower than users' estimate	No difference	No difference
Performance enhancing drug use	Yes	No difference	Higher than non-users' estimate	No difference
	No	No difference	Lower than users' estimate	No difference
Nutritional supplement use	Yes	No difference	*Ambiguous*	No difference
	No	No difference		No difference

## Method

Following ethics approval, an on-line survey collected data from students attending a South-Eastern Australian university. Every student of the student population of > 40,000 who logged into the website during the 4-week period in 2009 received the link to the survey, with no tracking option as imposed on the project by the institutional ethics committee. This convenience sampling method yielded 186 responses, of which n = 127 (86 usable) were athletes and n = 59 were from the general student population (comparison group for selected questions). A number of respondents provided variably incomplete data across the question set.

The athlete group (86 usable) was further restricted to those in 3^rd ^grade sport or above (n = 46) imposed for investigating the mental representation. This selection criterion was based on the level of sport involvement. Australian 3^rd ^grade sport represents a point at which club sport begins to feed into professional or elite sport, or serve as a transition after injury or is used to regain form for professional or elite athletes. Students who reported participating at 3^rd ^grade or above captures student athletes who may experience the competitive pressures of sport differently to those who participate in recreational or social sport. Recruitment was via a link on the top page of the administrative web site used by students for enrolment, examination timetables and so on. The on-line survey was part of a larger study on responses to the use of different kinds of substances in sporting contexts. Only the measures of relevance are reported, namely: i) age, gender, sport and level of participation; ii) questions on illicit drug use in the general population and personal use; iii) questions on ergogenic supplement use in the general population and personal use; and iv) questions on doping use in the athlete population and personal use.

The dependent variables were estimated population prevalence of nutritional supplement, illicit drug use and doping. These substances were defined as follows: '*Nutritional supplements*' are vitamins (e.g. vitamin A, B, C, E, D, etc.), minerals (e.g. iron, zinc, calcium, magnesium, etc.) and non-vitamin non-mineral substances including herbals and botanicals (e.g. creatine preparations, St John's Worth, Ginco biloba, Echinacea, Guarana, Ginseng, protein solutions, glucosamine, coenzyme Q10, lecithin, melatonin, fish oil, shark cartilage, etc.). '*Doping*' or '*banned substances*' are those substances that are prohibited by the World Anti-Doping Agency or other governing body in training and/or competition. '*Social drugs*' are psychoactive drugs (e.g. stimulants, opiates, cannabis, cocaine, etc.) used for recreational purposes rather than for work, medical or spiritual reasons. Although caffeine, alcohol and tobacco are also social drugs, are excluded from the definition in this survey. These definitions were provided at the start of the questionnaire and used in this manuscript. The terms 'social drugs' and 'illicit drugs'; 'doping' and 'prohibited performance enhancing substances' are used interchangeably throughout the paper.

The FCE was gauged by asking respondents to make an estimation of the level of fellow athletes using prohibited performance enhancing substances, illicit drugs and nutritional supplements independently asking '*What % of others in your sport has used a banned substance?*'. The responses were recorded on a percentage scale where 0% means that nobody and 100% means that everybody uses a substance.

In addition, athletes were asked about their beliefs regarding the effectiveness and necessity of various performance enhancing substances using the following questions: i) '*Do you believe that most people need supplementation to balance their diet?*' (Yes/No/I don't know) and ii) '*Do you believe that it is possible to win in high level sport competitions without doping?*' (Yes/No/I don't know).

The FCE tests compared estimated lifetime prevalence rates by personal use (independent variables with levels "yes" and "no"). Domain specificity was assessed by examining whether the FCE emerged by personal use within and across substances. In operational terms, domain specificity emerges from the absence of the FCE when users of one substance (e.g. illicit drug users) estimate the prevalence of another unaccepted drug (e.g. doping). Estimations for substances with similar ergogenic effects to doping but with generally accepted use (e.g. nutritional supplements) were expected to show no difference between users and non-users.

One-way analysis of variance assessed differences between groups with Tukey's HSD post hoc test when required. To test domain specificity, logistic regression was used for the prediction of the probability of an event (i.e., whether an athlete uses banned substances regularly) by fitting a logistic curve to a predictor variable [35]. Model fit was estimated using Akaike information criterion (AIC), defined as 2k - 2ln (*L)*, where *L *is the likelihood for an estimated model with *k *parameters. Thus the AIC is not testing the model with a traditional null hypothesis but rather, it affords ranking and comparing competing statistical models taking complexity into account. Statistical analyses were performed using PASW Statistics 17 and R Statistical Computing.

## Results

The mean age of the athletes in the sample (n = 86) was 23.07 (SD = 3.81; range 18-37; 53.5% female). The prevalence rates for self-reported nutritional supplement and social drug use are presented in Table 2. Prevalence rates for the general student population in the sample are presented for comparison. Only about one-third (27/86) of the athletes believed that most people need supplementation to balance their diet and 69/86 believed that winning without doping is possible even at a high level. These views suggest that in athletes' mind, supplementing with prohibited and/or acceptable 'extras' is not necessary. Whilst the majority of the athletes thought that prohibited performance enhancing substances are effective (ranging from effective to very effective), half of the respondents did not know if supplements are good and healthy substitutes for prohibited performance enhancements or not.

**Table 2:** Self reported illicit drug and nutritional supplement use in the sample (no doping use was reported)

	Illicit drug only	Supplements only	Both	None	Total
Student-athletes	24	17	13	32	86
Students^a^	12	12	2	19	45
Total	36	29	15	51	131

^a ^14 missing

Among athletes who responded to the survey, 46 met the criteria of sport participation 3^rd ^grade or above (average age = 23.62, SD = 3.93; range 18-37; 60.0% female; 1 missing). Self-reported supplement use (43.5%) was below that reported by the only epidemiological study on Australian supplement use (52.2%) [42]. Self-reported illicit drug use (47.8%) exceeded Australian general population lifetime use (38.1%) but was comparable to the illicit lifetime drug use in the 20-29 age group (54.0%) [43]. Some respondents (21.7%) reported using both supplements and illicit drugs, leading to four groups: non-users; supplement only; illicit only; and "both". No respondent self-reported doping. The official estimate of doping in Australia in 2009 was 1%; 23/2312 athletes in the Australian Registered Testing Pool were placed on the Register of Findings for performance enhancing substances in 2008-2009, excluding cannabinoids and administrative entries [44]. Aggregated lifetime prevalence estimates of use were approximately normally distributed for supplements (range 10-70, skewness = 0.07, kurtosis = -0.96), illicit drugs (range 10-99, skewness = -0.63, kurtosis = -0.35) and doping (range 0-50, skewness = 1.37, kurtosis = 1.07).

**Figure 2:**
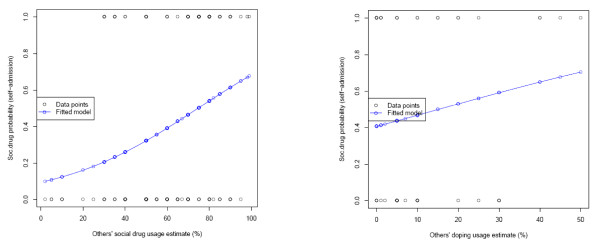
Prediction model fit within (A) and outside (B) domain of illicit drugs.

Evidence supporting domain specificity was found among self-admitted illicit drug users. The simple models of fitted logistic regression curves predicting the probability of social drug use based on social drug use estimate and doping use estimate are depicted in Figure 2.

The model based on self-reported illicit drug use and illicit drug use estimate showed a good fit (AIC = 179.180, -logL = 175.180, df = 137, p < 0.001); whereas the model for predicting doping use based on self-admitted social drug use was unsatisfactory (AIC = 66.494, -logL = 62.494, df = 44, p = 0.276). In practical terms, the model fit (or the lack of fit in the case of cross drug type prediction) provides some evidence for domain specificity. The social drug use estimations given by those who admitted its use were congruent with the previously observed FCE and resulted in a well fitting prediction model. On the contrary, social drug users' estimation of doping use failed to show an acceptable relationship between behaviour and estimation. Should there be no distinction made in athletes' minds about these two classes of drugs (social drug and doping), we would expect to see good fitting models across all four possible combinations of doping use, drug use, doping estimates and social drug use estimates.

As expected, differences in social projection by the user groups, including mixed groups, reflected the group characteristics. All groups overestimated population and sample prevalence of illicit drug use. No statistically distinct FCE emerged for supplement use. Means, standard deviations and ANOVA test statistics with corresponding p-values are presented in Table 3. Using the four "user" groups, the FCE was statistically demonstrated for illicit drug use, where post-hoc testing revealed the illicit only group overestimated prevalence relative to the non-user group. Supplement only users reflected non-users, and the "both" group sat between the other groups. Supplement only users appeared to realistically gauge supplement use, at least within the sample, with other groups underestimating. Illicit only users underestimated by the greatest degree, followed by "both" and non-users. No statistically distinct FCE emerged for doping estimates. Supplement users estimated doping 8-12 points less than all other groups. The "both" group overestimated to the greatest degree, with non-users and illicit only users offering similar average estimates. All groups overestimated the official population prevalence.

**Table 3:** Illicit drug, supplement and doping estimates by illicit and supplement drug use

Substance	User Status	n	Mean	SD	F	p
Illicit Drug	Non-User	14	50.71	19.20	3.55	<0.03
	Illicit Only	12	74.08	17.30		
	Supplement Only	10	55.20	23.56		
	Both	10	67.50	20.58		
	
						
	
Supplement	Non-User	14	38.21	13.39	2.23	>0.09
	Illicit Only	12	30.00	14.30		
	Supplement Only	10	46.30	18.05		
	Both	10	34.50	15.36		
	
						
	
Doping	Non-User	14	13.57	11.00	1.49	>0.23
	Illicit Only	12	12.25	15.45		
	Supplement Only	10	4.50	3.75		
	Both	10	16.10	17.82		

In terms of assessing the behavioural domain for doping, the results are ambiguous. Overestimation of doping by non-users suggests something other than substance use drove the estimation. The relative underestimation (although well above official estimates) by supplement users supports this notion. The pattern of doping estimation approximates that of illicit drugs with all groups overestimating prevalence. This pattern fails to provide firm evidence whether doping is related or unrelated to other substance use behaviours.

## Discussion

The FCE was found for illicit substance use and was evident as a trend for ergogenic supplement use. It is unclear whether the results point to a relationship between doping and either or neither of the other substances. The results associated with respondents who used supplements suggested that doping estimates may be influenced by ergogenic supplement use. Individuals who used supplements tend to inflate the percentage of individuals who dope but to a much smaller degree than those who use other substances. In addition to these main results, illicit drug use and doping were overestimated. This indicates that, self-report notwithstanding; Australian university athletes may have unrealistic perceptions of illicit drug use and doping [40]. While the pilot nature of this study, especially the small sample, curtails generalisability. The results are therefore interpreted under a generalisability caveat and are intended to inform the broader research program with regard to the observed trends.

The presence of the FCE within rather than between substances provides an indication that nutritional supplement use and illicit drug use come from different behavioural domains. Individuals who admitted using one particular type of drug tend to inflate the percentage of individuals who uses the same drug to a much larger degree than those who use other substances. This suggests the FCE could provide an expedient way of identifying when interventions designed to influence one behaviour could influence another. The results indicate that interventions aimed at illicit drug use are unlikely to have much effect on supplement use, and *vice versa*.

Estimates of those who used both supplements and illicit drugs were more akin to illicit drug users, suggesting illicit drug use may be a dominant behavioural domain. As Table 3 shows, users of 'both' illicit drugs and NS gave similar social projections (67%) for illicit drug use to the projected figure of those who use illicit drugs only (74%), compared to a definitely lower estimate (55%) given by NS-only users, suggesting that users of 'both' may have behaved like illicit drug users due to that domain requiring a different psychological mechanism. For example, users of both may do so as a function of substance use, whereas supplement only users may do so for ergogenic reasons. The dominance of illicit drug use may have implications for the domain specificity of doping behaviour. A reversed pattern was observed for NS use projection, where NS-only users gave higher estimation (46%) compared to those who use both (34%), thus providing further evidence that in mixed behavioural categories, and the self-anchored behavioural domain is context specific.

The effort to determine whether doping was more akin to illicit drug or ergogenic supplement use led to mixed results. Out of these mixed results, the marked lower estimate by supplement users may be a clue. Mazanov *et al *[13] showed that elite athlete supplement users were more knowledgeable about anti-doping rules and procedures than non-users. Extrapolating this observation to prevalence, athlete supplement users may be more knowledgeable about prevalence rates compared to those who have no contact with ergogenic substances. The other estimates imply that illicit drug users (including the 'both' group) are as ignorant as non-users when it comes to doping. The hypothesised relationship between knowledge of doping and the anti-doping approach suggesting that doping is an ergogenic rather than a substance use phenomenon needs to be tested.

The observation that the sample overestimated illicit drug and doping use is concerning. Such overestimation has been observed elsewhere [45] and represents a potential normative misperception among tertiary level students. The danger comes with such misperception being identified as a predictor of drug use [36,37], potentially increasing the likelihood of illicit drug use or doping. The misperception may reflect a bias in the overestimation of rare events [46,47]. While 38.1% of Australians report lifetime use, the proportion of users over different periods means illicit drug use is a relatively rare behaviour: 5.1% in the last week, 7.7% in the last month, 13.1% in the last 12 months [43]. Rarity is even more significant for doping given the low official rate. This issue could be assessed by calibrating FCE studies against behaviours with different prevalence rates, such as universal, common, uncommon and rare. The result also underscores the need to generate realistic norms around illicit drug use and doping use among university student athletes.

The sample sizes themselves are a clear indication of the low statistical power. In this instance, the statistical tool is used to establish direction for future research rather than establish definitive results that require a higher level of statistical integrity. The intention of this short report was to draw researchers' and policy makers' attention to this potentially important aspect of doping behaviour to facilitate further targeted research. Future research building on this pilot study should involve collecting data from a representative larger sample that delivers statistical certainty. Assessing knowledge of doping and anti-doping to determine if it impacts doping estimates would strengthen the findings.

## Conclusions

This pilot study provided an indication that harnessing the FCE might be a fruitful avenue to further examine whether doping behaviour has more in common with illicit drug use or ergogenic supplement use. The pilot nature of the study suggests that doping may be an ergogenic phenomenon, however further testing with an improved research design and sample is needed to establish any such claim. The importance of having a precise picture of the mental representation of doping is underscored by the increasing need for effective anti-doping prevention and intervention. Further research is required to establish if some athletes project their own behavioural tendencies or actual behavior onto other athletes and assume that many others feel or do the same and indeed are using prohibited performance enhancing substances. As a consequence, their own doping tendency or behaviour appears normal and normative, so that they can follow it without compromising their own self-esteem and social acceptance. In this vein, FCE has importance beyond being a useful vector to understanding the position of doping in athletes' minds. These considerations, coupled with the mental representation of doping in athletes' minds, suggest possible intervention strategies to increase compliance with anti-doping initiatives.

## Competing interests

The authors declare that they have no competing interests.

## Authors' contributions

AP drafted and finalised the paper, and with DPN designed the study and the questionnaire. JM collected and analysed the data, and developed early drafts. All authors approved the final version the paper.
